# Supplementary material for: Estrogen-related receptor gene expression associates with sex differences in cortical atrophy in isolated REM sleep behavior disorder
Source: Nat Commun. 2025 Oct 10;16:9016. doi: 10.1038/s41467-025-63829-w (PMC12514017; doi:10.1038/s41467-025-63829-w)
Supplement: Supplementary file 2 — Reporting Summary [file 41467_2025_63829_MOESM2_ESM.pdf]

Corresponding author(s): Marie Filiatrault, Dr Shady Rahayel

Last updated by author(s): Aug 25, 2025

Reporting Summary

Nature Portfolio wishes to improve the reproducibility of the work that we publish. This form provides structure for consistency and transparency in reporting. For further information on Nature Portfolio policies, see our Editorial Policies and the Editorial Policy Checklist.

Statistics

For all statistical analyses, confirm that the following items are present in the figure legend, table legend, main text, or Methods section.

n/a	Confirmed	
☐	☒	The exact sample size (*n*) for each experimental group/condition, given as a discrete number and unit of measurement
☐	☒	A statement on whether measurements were taken from distinct samples or whether the same sample was measured repeatedly
☐	☒	The statistical test(s) used AND whether they are one- or two-sided<br>*Only common tests should be described solely by name; describe more complex techniques in the Methods section.*
☐	☒	A description of all covariates tested
☐	☒	A description of any assumptions or corrections, such as tests of normality and adjustment for multiple comparisons
☐	☒	A full description of the statistical parameters including central tendency (e.g. means) or other basic estimates (e.g. regression coefficient) AND variation (e.g. standard deviation) or associated estimates of uncertainty (e.g. confidence intervals)
☐	☒	For null hypothesis testing, the test statistic (e.g. *F*, *t*, *r*) with confidence intervals, effect sizes, degrees of freedom and *P* value noted<br>*Give P values as exact values whenever suitable.*
☐	☒	For Bayesian analysis, information on the choice of priors and Markov chain Monte Carlo settings
☐	☒	For hierarchical and complex designs, identification of the appropriate level for tests and full reporting of outcomes
☐	☒	Estimates of effect sizes (e.g. Cohen's *d*, Pearson's *r*), indicating how they were calculated

Our web collection on statistics for biologists contains articles on many of the points above.

Software and code

Policy information about availability of computer code

Data collection	No commercial, open-source or custom codes were used to collect the data in this study.
Data analysis	MRI scans were processed to generate whole-brain vertex-based cortical surface maps using FreeSurfer (version 7.1.1). For the brain parcellation, we used netneurotools.datasets.fetch_cammoun2012 in python (doi: 10.1016/j.jneumeth.2011.09.031) to retrieve atlas annotation files in fsaverage5 template space, which were then registered to each participant’s cortical surface via FreeSurfer’s mri_surf2surf function. The aligned annotation files were converted to gifti format using mris_convert for compatibility with downstream processing, and cortical thickness measures were extracted for each parcellated region. To account for scanner-specific effects in this multicentric dataset, cortical thickness values were harmonized across acquisition sites using the ComBat tool in MATLAB (https://github.com/Jfortin1/ComBatHarmonization). W-scoring was done in MATLAB to remove age and sex effects in normal aging, as explained in other work (Tremblay et al., 2020). Gene expression microarray data was accessed using abagen in python (version 0.1.3), following recommendations for preprocessing and normalization (https://github.com/rmarkello/abagen). We applied partial least squares (PLS) regression in MATLAB to determine whether regional gene expression patterns were associated with sex effects on cortical atrophy in iRBD. Brain regions were randomly shuffled using a spherical reassignment procedure that preserved spatial autocorrelation, described at https://github.com/frantisekvasa/rotate_parcellation/. Gene set enrichment analysis was done using the WebGestalt 2024 web portal (https://www.webgestalt.org/). Gene expression from the GTEx Project were accessed online through the web portal (https://gtexportal.org/home/aboutAdultGtex), specifically the GTEx Analysis Release V10 (dbGAP Accession phs000424.v10.p2).

For manuscripts utilizing custom algorithms or software that are central to the research but not yet described in published literature, software must be made available to editors and reviewers. We strongly encourage code deposition in a community repository (e.g. GitHub). See the Nature Portfolio guidelines for submitting code & software for further information.

Data

Policy information about availability of data

All manuscripts must include a data availability statement. This statement should provide the following information, where applicable:

- Accession codes, unique identifiers, or web links for publicly available datasets
- A description of any restrictions on data availability
- For clinical datasets or third party data, please ensure that the statement adheres to our policy

The imaging and clinical data used in this study were obtained from multiple collaborating centres, each of which retains ownership of their respective datasets. The principal investigator had authorized access to all data necessary for the analyses performed in this study. Access to the data is restricted due to institutional policies and participant privacy regulations. Requests for data access must be submitted directly to the respective data-holding institutions and are subject to their local ethical and legal frameworks. These restrictions limit the sharing of raw data in a public repository. Access requests should clearly specify the intended purpose of use and may be granted to qualified researchers following institutional approval. The expected timeframe for review of such requests may vary depending on the institution. Source data generated and analyzed during this study, where permitted, are provided with this paper.

Research involving human participants, their data, or biological material

Policy information about studies with human participants or human data. See also policy information about sex, gender (identity/presentation), and sexual orientation and race, ethnicity and racism.

Reporting on sex and gender	In this work, we investigated the effects of sex on brain atrophy in isolated REM sleep behavior disorder. The sex of participants was assigned by the clinician (female or male). We only considered biological sex (female/male) as the data collected from every site do not allow, at the moment, to disentangle the effects of sex from gender.We therefore always refer to sex labels such as female and male. Differences in clinical features between females/women and males/men with synucleinopathies have been documented, but there are still no studies having investigated brain changes in relation to sex in isolated REM sleep behavior disorder.
Reporting on race, ethnicity, or other socially relevant groupings	n/a, not available from the assembled cohorts.
Population characteristics	This cohort included 408 polysomnography-confirmed iRBD patients and 480 age-matched healthy controls, recruited from 9 international centers. All iRBD patients underwent video-polysomnography and were diagnosed based on the International Classification of Sleep Disorders criteria. Neurological evaluations and cognitive assessments confirmed that patients were still in the isolated phase of RBD. All patients underwent standardized clinical evaluations, including motor assessments using the Movement Disorders Society-sponsored Unified Parkinson's Disease Rating Scale (MDS-UPDRS-III), global cognitive evaluation using the Montreal Cognitive Assessment (MoCA) and assessment of olfactory identification performance. Each cohort underwent either the 12-item Sniffin' Sticks, the 16-item Sniffin' Sticks, the 40-item University of Pennsylvania Smell Identification Test (UPSIT-40), or the reduced 12-item version of the UPSIT (UPSIT-12). Of the 888 eligible participants (408 iRBD patients and 480 controls), 7 (0.8%) did not pass processing (4 iRBD patients, 3 controls) and 134 (15.1%) did not pass quality control (61 iRBD patients, 73 controls). At this point groups differed in age, which led to the exclusion of 60 controls under 54 years old, resulting in a final sample of 687 participants for analysis: 343 iRBD patients and 344 healthy controls. The iRBD group comprised 49 (14%) females and 294 (86%) males, while the control group included 131 (38%) females and 213 (62%) males. Groups did not differ in age, iRBD were around 67 years old (+/- 6.9) and controls were around 66.6 years old (+/- 6.9).
Recruitment	A total of 888 participants were recruited for this study and underwent T1-weighted brain MRI imaging. This cohort included 408 polysomnography-confirmed iRBD patients and 480 age-matched healthy controls, recruited from nine international centers: 179 (85 patients) from the Centre for Advanced Research on Sleep Medicine at the Hôpital du Sacré-Cœur de Montréal and The Neuro (controls from Quebec Parkinson Network), Montreal, Canada; 140 (83 patients) from the First Faculty of Medicine at Charles University, Prague, Czechia; 147 (81 patients) from the Oxford Discovery Cohort, Oxford, UK; 136 (60 patients) from the Movement Disorders Clinic (ICEBERG and ALICE cohorts) at the Hôpital de la Pitié-Salpêtrière, Paris, France; 56 (30 patients) from the ForeFront Parkinson's Disease Research Clinic, Sydney, Australia; 38 (18 patients) from Aarhus University Hospital, Aarhus, Denmark; 29 (14 patients) from IRCCS Ospedale Policlinico San Martino, Genoa, Italy; and 163 (37 patients) from the Parkinson's Progression Markers Initiative (PPMI) baseline cohort.
Ethics oversight	All participants provided written informed consent. Study protocols were approved by the Research Ethics Board of the Quebec Integrated University Centre for Health and Social Services of Northern Island of Montreal (MEO-37-2024-2699), the McGill University Health Centre (MP-37-2022-7744), and the respective local ethics boards at all participating sites.

Note that full information on the approval of the study protocol must also be provided in the manuscript.

Field-specific reporting

Please select the one below that is the best fit for your research. If you are not sure, read the appropriate sections before making your selection.

☒ Life sciences ☐ Behavioural & social sciences ☐ Ecological, evolutionary & environmental sciences

For a reference copy of the document with all sections, see nature.com/documents/nr-reporting-summary-flat.pdf

Life sciences study design

All studies must disclose on these points even when the disclosure is negative.

Sample size	Those are convenience sample coming from several ongoing large prospective studies. They represent the total amount of participants available for those analyses.
Data exclusions	Patients were excluded if they had, at the clinical visit closest in time to the MRI session, a diagnosis of DLB, PD or MSA based on published diagnostic criteria, had a history of brainstem stroke, epilepsy or epileptiform activity on EEG, had antidepressant-triggered RBD, had untreated obstructive sleep apnoea, or had RBD mimics such as sleepwalking and night terrors.
Replication	This is the largest dataset available so far, there is no other existing dataset as large as the one used for this study.
Randomization	Randomization was not needed, the grouping for the analyses was based on comparing iRBD patients to control participants.
Blinding	Blinding for visual quality control of MRI scans

Reporting for specific materials, systems and methods

We require information from authors about some types of materials, experimental systems and methods used in many studies. Here, indicate whether each material, system or method listed is relevant to your study. If you are not sure if a list item applies to your research, read the appropriate section before selecting a response.

Materials & experimental systems

n/a	Involved in the study	
☒	☐	Antibodies
☒	☐	Eukaryotic cell lines
☒	☐	Palaeontology and archaeology
☒	☐	Animals and other organisms
☒	☐	Clinical data
☒	☐	Dual use research of concern
☒	☐	Plants

Methods

n/a	Involved in the study	
☒	☐	ChIP-seq
☒	☐	Flow cytometry
☐	☒	MRI-based neuroimaging

Plants

Seed stocks	n/a
Novel plant genotypes	n/a
Authentication	n/a

Magnetic resonance imaging

Experimental design

Design type	n/a
Design specifications	n/a
Behavioral performance measures	n/a

Acquisition

Imaging type(s)	Structural
Field strength	3T
Sequence & imaging parameters	T1-weighted MRI scans were acquired across sites using 3T MRI scanners with varying acquisition protocols according to the research center. In the Montreal cohort, scans were obtained using a 3T Siemens TIM Trio scanner with 12-channel

head coil and an MPRAGE sequence (TR = 2300 ms, TE = 2.91 ms, flip angle = 9°, isotropic voxel size = 1 mm) or a 3T Siemens PRISMA scanner with a 32-channel head coil and an MPRAGE sequence (TR = 2300 ms, TE = 2.98 ms, flip angle = 9°, isotropic voxel size = 1 mm). The Oxford cohort used a 3T Siemens Trio MRI Scanner with a 12-channel head coil and an MPRAGE sequence (TR = 2040 ms, TE = 4.7 ms, flip angle = 8°, isotropic voxel size = 1 mm). The Prague cohort used a 3T Siemens Skyra with a 32-channel head coil and an MPRAGE sequence (TR = 2200 ms, TE = 2.4 ms, flip angle = 8°, isotropic voxel size = 1 mm). The Paris cohort used a 3T Siemens TIM Trio scanner with a 12-channel head coil and an MPRAGE sequence (TR = 2300 ms, TE = 4.18 ms, flip angle = 9°, isotropic voxel size =1 mm) or a 3T PRISMA Fit scanner with a 64-channel head coil and an MP2RAGE sequence (TR = 5000 ms, TE = 2.98 ms, flip angles = 4° and 5°, GRAPPA = 3, isotropic voxel size = 1 mm). The Sydney cohort used a GE Discovery MR750 3T scanner with an 8-channel head coil and a BRAVO sequence (TR = 5800 ms, TE = 2.6 ms, flip angle = 12°, isotropic voxel size = 1 mm). The Aarhus cohort used a 3T Siemens MAGNETOM Skyra scanner with a 32-channel head coil and an MPRAGE sequence (TR = 2420 ms, TE = 3.7 ms, flip angle = 9°, isotropic voxel size = 1 mm). The Genoa cohort used a 3T Siemens PRISMA Scanner with a 64-channel head coil and an MPRAGE sequence (TR = 2300 ms, TE = 2.98 ms, flip angle = 9°, isotropic voxel size = 1 mm). The acquisition parameters for the PPMI study have been described elsewhere.

Area of acquisition: Whole brain scan were acquired for all cohorts and analyzed

Diffusion MRI: ☐ Used ☒ Not used

Preprocessing

Preprocessing software: Cortical reconstruction and volumetric segmentation was performed with the Freesurfer image analysis suite version 7.1.1, which is documented and freely available for download online (http://surfer.nmr.mgh.harvard.edu/). The technical details of these procedures are described in prior publications (Dale et al., 1999; Dale and Sereno, 1993; Fischl and Dale, 2000; Fischl et al., 2001; Fischl et al., 2002; Fischl et al., 2004a; Fischl et al., 1999a; Fischl et al., 1999b; Fischl et al., 2004b; Han et al., 2006; Jovicich et al., 2006; Segonne et al., 2004, Reuter et al. 2010, Reuter et al. 2012). Briefly, the processing pipeline includes automated Talairach transformation, segmentation of the subcortical white matter and deep gray matter volumetric structures (including hippocampus, amygdala, caudate, putamen, ventricles) (Fischl et al., 2002; Fischl et al., 2004a) intensity normalization (Sled et al., 1998), tessellation of the gray matter white matter boundary, automated topology correction (Fischl et al., 2001; Segonne et al., 2007), and surface deformation following intensity gradients to optimally place the gray/white and gray/cerebrospinal fluid borders at the location where the greatest shift in intensity defines the transition to the other tissue class (Dale et al., 1999; Dale and Sereno, 1993; Fischl and Dale, 2000). Surface maps were smoothed with a 15-mm full-width half maximum kernel.

Normalization: The processing pipeline used automated Talairach transformation.

Normalization template: Automated Talairach registration procedure developed and distributed by the Montreal Neurological Institute (Talairach and Tournoux, 1988; Collins et al., 1994) was performed with the Freesurfer processing pipeline, to compute the transformation matrix from our T1-weighted scans.

Noise and artifact removal: The processing pipeline included motion correction (Reuter et al. 2010), removal of non-brain tissue using a hybrid watershed/surface deformation procedure (Segonne et al., 2004)

Volume censoring: n/a

Statistical modeling & inference

Model type and settings: Group differences in continuous demographic and clinical variables were assessed using independent sample t-tests for normally distributed variables, and with non-parametric Mann-Whitney U test for non-normally distributed variables. Categorical variables were compared using chi-squared tests. Vertex-based cortical thickness analyses were conducted in FreeSurfer using general linear models to assess sex-by-group interactions on cortical thickness, surface area, and volume, adjusting for age and acquisition site. For surface area and cortical volume analyses, estimated total intracranial volume (eTIV) was included as an additional covariate. For the analysis of subcortical volumes, volumes were also normalized by the eTIV.

Effect(s) tested: Independent sample t-tests for demographics and clinical variables normally distributed, and non-parametric Mann-Whitney U test for non-normally distributed variables. Sex-by-group interaction (ANOVA) for vertex-based cortical thickness, volume and surface area analyses.

Specify type of analysis: ☒ Whole brain ☐ ROI-based ☐ Both

Statistic type for inference (See Eklund et al. 2016): Cluster-level statistical threshold of $P < 0.05$. Vertex-level significance set at $P < 0.05$.

Correction: Cluster-level significance was determined using Monte Carlo spatial permutations, FDR correction was applied to control for multiple comparisons in the parcel-wise analyses.

Models & analysis

n/a	Involved in the study
☒	☐ Functional and/or effective connectivity
☒	☐ Graph analysis
☒	☐ Multivariate modeling or predictive analysis
